# Effects of Replacing Cottonseed Meal with Corn Dried Distillers’ Grain on Ruminal Parameters, Performance, and Enteric Methane Emissions in Young Nellore Bulls Reared in Tropical Pastures

**DOI:** 10.3390/ani11102959

**Published:** 2021-10-14

**Authors:** Tiago Luís Da Ros de Araújo, Wilton Ladeira da Silva, Andressa Scholz Berça, Abmael da Silva Cardoso, Rondineli Pavezzi Barbero, Eliéder Prates Romanzini, Ricardo Andrade Reis

**Affiliations:** 1 Department of Animal Sciences, São Paulo State University, Jaboticabal 14884-900, Brazil; tiago.luisaraujo@zootecnista.com.br (T.L.D.R.d.A.); abmael.cardoso@unesp.br (A.d.S.C.); elieder.romanzini@gmail.com (E.P.R.); ricardo.reis@unesp.br (R.A.R.); 2 Department of Animal Sciences, Federal University of Goiás, Goiânia 74690-900, Brazil; wiltonladeira@ufg.br; 3 Department of Animal Sciences, Federal Rural University of Rio de Janeiro, Seropédica 23890-000, Brazil; barbero.rp@gmail.com

**Keywords:** continuous grazing, *Urochloa brizantha*, supplementation, tropical grass, greenhouse gases

## Abstract

**Simple Summary:**

Dried distillers’ grains (DDG), a co-product of ethanol production from corn, is gaining attention for its efficiency in ruminant nutrition, as it meets both the energy and protein demands of livestock and is less costly than the popular alternatives. The aim of this study was to evaluate the effect of replacing cottonseed meal with DDG at two levels (50 and 100%) on young Nellore bulls grazing Marandu grass in the rainy season, focusing on ruminal parameters, animal performance, and methane (CH_4_) emissions. When replacing 50% of cottonseed meal with DDG, animals presented higher intakes of dry matter, organic matter, forage, and digestible organic matter, compared to 100% DDG. Ruminal parameters, including pH, acetate, and acetate: propionate, were higher when animals received only mineral supplementation. Animals supplemented with concentrate (cottonseed meal and/or DDG) presented greater daily weight gain and final body weight than the animals consuming mineral supplementation. Replacing cottonseed meal with DDG does not cause great variations in ruminal parameters, animal performance, and enteric CH_4_ emissions in grazing Nellore cattle during the rearing phase in the wet season. However, supplementation of 0.3% body weight with the concentrate can improve the productive performance of grazing animals. Both protein sources, DDG or cottonseed meal, can be used to intensify grazing beef cattle production.

**Abstract:**

Two experiments were conducted to evaluate the effect of replacing cottonseed meal with DDG on ruminal parameters, methane (CH_4_) emissions (Experiment 1), and animal performance (Experiment 2) of young Nellore bulls grazing Marandu grass during the rainy season. Four supplementation strategies were used in both experiments: (1) Mineral supplementation (MS); (2) conventional multiple supplement (energy/protein) with cottonseed meal and citrus pulp (CMS); (3) CMS with 50% cottonseed meal replaced by DDG (50DDG); and (4) CMS with 100% cottonseed meal replaced by DDG (100DDG). The 50DDG condition resulted in greater intake of dry matter (*p* = 0.033), organic matter (OM) (*p* = 0.050), forage (*p* = 0.035), and digestible OM (*p* = 0.031) than 100DDG. The supplemented animals presented greater final body weight (BW) and average daily gain than the animals consuming MS (*p* = 0.011), and lower pH, acetate, and acetate:propionate (*p* < 0.05). However, the treatments had no influence on stocking rate, gain per area, and enteric CH_4_ emissions (*p* > 0.05). Replacing cottonseed meal with DDG does not result in great variations in ruminal parameters, animal performance, and enteric CH_4_ emissions of grazing Nellore cattle during the rearing phase in the wet season. Both protein sources in 0.3% BW supplementation can be used to intensify beef cattle production in pastures.

## 1. Introduction

In order for Brazilian beef cattle farming to be consolidated in sustainable livestock intensification (SLI) and meet the demands of global markets, it should promote the efficient use of resources whilst reducing negative environmental impacts. However, this intensification has led to increasing the competition between cattle and humans for food sources. 

Without SLI, the national livestock will sustain low production rates across approximately 162 mha of pasture in 2020 [1]. Degraded pastures represent 50–60% of the total area utilized in Brazil for cattle production, which negatively impacts the environment, mainly due to greenhouse gas emissions, deforestation, and soil degradation [2]. In this scenario, supplementation with inedible human feed can reduce costs, increase productivity, and improve the efficiency of land utilization [3,4].

In Brazil, mineral supplementation with macro- and micro-nutrients is necessary due to the low mineral content of soil, particularly phosphorus [5]. In addition, the level of phosphorus in tropical grasses is low, and the fertilization of pastures only aims to meet the critical levels of plants due to the high cost of fertilizers. Therefore, mineral supplementation in feed is required [5].

On the other hand, less costly sources of animal feed that are not consumed by humans have gained increasing attention, for example, citrus pulp as an energy source and dried distiller’s grain (DDG) as a protein source [3,6]. Cottonseed meal is a traditional protein source used in ruminant nutrition, mostly during the rearing and finishing phases, owing to its high ruminal undegraded protein (RUP) content [7,8], which can lead to an increase in protein flow to the small intestine. 

An alternative source of protein to cottonseed meal is DDG, a co-product of the corn or sorghum ethanol industry, which has recently emerged in Brazil as a competitively priced beef cattle supplement, mainly in the Midwest region of the country [9]. Moreover, DDG has a high protein concentration when dried, high digestibility, and high RUP, making it a potential substitute for more expensive ingredients used in animal and human nutrition, such as soybean. However, information on the chemical composition and nutritive value of DDG produced in Brazil is still scarce, mainly due to the heterogeneity of its manufacturing processes. Most of the studies on this co-product were conducted in the USA and Canada, which produce soluble DDGs, whilst in Brazil, many manufacturers produce non-soluble DDGs.

According to Omer et al. [10], DDG can replace 10 to 50% of cottonseed meal during the rearing phase of crossbred young bulls receiving 2% BW supplementation, as it improves digestibility, performance, and economic efficiency. Hoffmann et al. [8] observed that corn DDG can replace 100% of cottonseed meal as a protein source during the rearing phase in tropical pastures without any adverse effects on average daily gain (ADG), enteric methane (CH_4_) emissions or nitrogen (N) excretion. Moreover, Hoffmann et al. [6] found that the use of DDG does not affect the performance of animals finished on pasture or feedlot, and that this co-product is a viable alternative to replace conventional supplements in tropical environments. However, the efficiency of different replacement levels of cottonseed meal by DDG, and the responses of animals grazing tropical pastures has not yet been described. 

Supplementation with concentrates is a common practice used in intensive systems to improve animal performance. It is an alternative for CH_4_ mitigation, as it reduces rumen pH, alters the acetate: propionate ratio, and decreases the amount of CH_4_ produced per unit of feed intake as the level of supplementation increases [11]. Therefore, suitable practices of supplementation and pasture management can decrease CH_4_ emissions [12,13] by reducing the intake of poorly digestible fiber, and the time of animals in the production system.

Research has shown beneficial effects on enteric CH_4_ emissions from young animals receiving DDG in diets, which can reach 16% [8,14]. Hünerberg et al. [15] reported a decrease of 12.6, 18, and 20% in CH_4_ emissions per g/d, g/kg of dry matter, and % of gross energy intake, respectively, when animals received diets with DDG *ad libitum* compared to the control diet (87% barley grain). Nevertheless, most of the previous studies on the effects of DDG on animal performance and environmental impacts of beef cattle production have been conducted in temperate regions and using DDGs and diets with higher levels of concentrates. Therefore, information on the effects of supplementation with DDG on Nellore performance and CH_4_ emissions in tropical environments is lacking. Knowledge of human inedible potential ingredients replacing traditional sources in cattle supplements can drive a more sustainable livestock system. 

We hypothesized that replacing cottonseed meal with DDG in supplements during the rainy season would not alter the ruminal parameters, performance, and CH_4_ emissions of beef cattle in tropical pasture. Therefore, the objective of this study was to evaluate the effects of replacing cottonseed meal with DDG on these outputs in young Nellore bulls grazing Marandu grass during the rainy season.

## 2. Materials and Methods

### 2.1. Experimental Area and Treatments

The two experiments were conducted in the Forage and Grasslands sector of the Sao Paulo State University “Julio de Mesquita Filho” (FCAV/UNESP), Jaboticabal, SP, located at 21°15′22″ South, 48°18′58″ West, at 595 m of altitude, during the wet season (January to May) of 2015. In Experiment 1, we evaluated intake, digestibility, and ruminal parameters. In Experiment 2, animal performance and CH_4_ emissions were evaluated. 

All of the research procedures were approved by the Ethics Committee of Sao Paulo State University (protocol code 12703/15).

According to the Department of Exact Sciences of FCAV/UNESP, the average precipitation during 2015 was 1424 mm and the mean temperature was 22.3 °C. The local climate is AW subtropical, with dry winters as well as warm and rainfall summers. The soil was classified as Ferralsol [4].

The experimental area consisted of 13.5 ha of *Urochloa brizantha* (Hochst. ex A. Rich.) R.D. Webster cv. Marandu (Marandu grass) sowed in 2001 and divided into 12 paddocks. In November 2014, we conducted a soil chemical analysis, and its results were as follows: pH 5.2; 28.0 g/dm^3^ of organic matter; 22.9 mg/dm^3^ of phosphorus, 18.0 mmolc/dm^3^ of calcium, 10.3 mmolc/dm^3^ of magnesium, and 57.0% base saturation. 

In December 2014, maintenance fertilization was performed in all of the paddocks with 50 kg P_2_O_5_ and 70 kg K_2_O/ha. Nitrogen fertilization was performed with 180 kg N/ha/year, using urea, divided into three equal applications, according to the precipitation schedule (January, February, and March 2015). 

The treatments consisted of four supplementation strategies in both experiments: (1) Mineral supplementation (MS) as a control treatment offered *ad libitum*; (2) conventional multiple supplement (energy/protein) with cottonseed meal (protein) and citrus pulp (energy) (CMS); (3) CMS with 50% cottonseed meal replaced with DDG (50DDG); and (4) CMS with 100% cottonseed meal replaced with DDG (100DDG) (Table 1). 

The diets were formulated according to the National Research Council (NRC) [7]. The 0.3% BW level of supplementation (CMS, 50DDG, and 100DDG) was chosen based on previous studies conducted by our research group, which reported that this level meets the animals’ requirements for weight gain (approximately 1.0 kg/day) and does not affect forage intake. In addition, this study aimed to evaluate the potential of supplements formulated with co-products of human food processing to meet the animals’ nutritional requirements, and the environmental, economic, and food security implications of their use. The inclusion of corn DGG was determined based on crude protein, and its chemical composition is presented in Table 2.

### 2.2. Pastures and Animal Management

In Experiment 1, we used eight young (14 ± 2-month-old) Nellore (*Bos taurus indicus*) bulls, rumen cannulated, with an average initial BW of 300.0 ± 27.0 kg. In Experiment 2, 60 young Nellore bulls with an initial BW of 345 ± 39 kg were identified, weighed, and randomly distributed into fixed groups of five animals (test) per paddock, adjusted according to BW. Then, of these 60 animals, 24 animals were randomly selected from the 12 paddocks (two animals/paddock) to evaluate enteric CH_4_ emissions and nutrient intake.

Pastures were managed under continuous stocking with a variable stocking rate, following the put and take technique [16], in order to maintain 25 cm of height. The height/forage mass relationship (bulk density) was evaluated weekly to estimate the herbage mass and adjust the stocking rate using the put and take technique, with reserve animals (regulators) from another herd, in addition to the five fixed test animals in the paddock. Under these experimental conditions, a previous study showed that 25 cm promotes 95% light interception, which allows maximum net forage accumulation to be achieved, with high leaf production and low senescence, which may increase animal performance [8,12,17]. 

The forage characteristics and chemical composition are presented in Table 3.

### 2.3. Experiment 1: Ruminal Parameters, Nutrient Intake, and Digestibility 

The cannulated animals were distributed in a double Latin square design, and four evaluation periods of 21 days (d) each were used. The animals were adapted to the diet for the first 16 d, and the last 5 d of the period were used to determine ruminal pH, ammoniacal nitrogen (N-NH_3_), short-chain volatile fatty acids (VFA), intake of forage and supplement, and digestibility.

The cannulated animals were kept in the same paddocks as the performance animals, with two animals in each treatment, and a supplement was provided daily at 11 am in the uncovered feeder, according to the respective treatment strategy and proposed Latin square. The cannulated animals were weighed at the beginning of the experiment after a 14 h feed and water fast, and every 21 d without fasting in order to adjust the supplement quantity and stocking rate. 

#### 2.3.1. Ruminal Parameters 

To evaluate the pH, N-NH_3_, and VFA, samples of ruminal liquid were collected from the cannulated animals in the last 2 days of each experimental period at time 0 (moment before supplementation), 2, 4, 6, and 8 h after supplementation. The ruminal liquid was collected at the solid-liquid interface and immediately filtered in a triple layer of gauze for pH measurement with a pH digital analyzer, calibrated with pH 7.0 and 4.0 buffers. One milliliter of sulfuric acid (1:1) was added to a sample of 50 mL of ruminal liquid and stored at −20 °C for subsequent analysis of N-NH_3_ and short-chain fatty acids. 

N-NH_3_ (mg/dL) was determined according to the Kjeldahl method [18]. The short-chain volatile fatty acid analysis was performed on a Shimadzu high-performance liquid chromatography (HPLC) system (model Prominence) equipped with an ultraviolet detector model SPD-20 and programmed to operate at a wavelength of 210 nm. The ruminal liquid samples were centrifuged at 7000 rpm for 5 min and filtered through a 13 mm diameter nylon filter (0.22 μm pore size). The injection volume was 20 μL. The quantified VFAs were acetate, propionate, and butyrate (mmol/L) [19]. 

#### 2.3.2. Nutrient Intake and Digestibility 

The forage intake by animals was determined based on individual fecal production, using chromium oxide (Cr_2_O_3_) as an external marker and indigestible neutral detergent fiber (iNDF) as an internal marker. 

For this assay, 10 g of Cr_2_O_3_/animal/d was provided to each animal at 9 a.m. for 10 d, the first 7 d for adaptation and the last 3 d for feces collection [20]. In the cannulated animals, Cr_2_O_3_ was directly administered into the rumen in a paper package and this marker started on the eleventh day of each experimental period, with the feces collection occurring in the last 3 d. For CH_4_-test animals, Cr_2_O_3_ was administered to each animal via the esophagus with a specific applicator in order to estimate the CH_4_ production per unit of dry matter intake. The feces were collected at 7 a.m. and 1 p.m. on the first day, at 9 a.m. and 3 p.m. on the second day, and at 11 a.m. and 5 p.m. on the third day. 

The fecal samples were identified, weighed, dried in an oven at 55 °C for 72 h, and then ground in a Wiley mill (Wiley Mill, Thomas Scientific, Swedesboro, NJ, USA) with a 1 mm sieve. All of the collections formed composite samples for each animal per period. Fecal recovery of Cr_2_O_3_ was determined using atomic absorption spectrophotometry [21]. From these data, Equation (1) was used to calculate fecal excretion [22].

(1) $$\mathrm{FE}\;\left(g/d\right)=\frac{{\mathrm{Cr}}_{2}O_{3}\;\mathrm{provided}\;\left(g/d\right)}{{\mathrm{Cr}}_{2}O_{3\;}\mathrm{concentration}\;\left(g/\mathrm{kg}\;\mathrm{DM}\right)}\;$$

The iNDF of forage, concentrates, and feces was determined by an in situ incubation procedure for 288 h [23]. After this period, the bags were adequately washed and dried at 55 °C for 72 h in an oven. The neutral detergent extraction in an Ankom fiber analyzer was performed in sequence, and the drying process was repeated for iNDF quantification [23].

From these values, the nutrient intake and digestibility were calculated (Equations (2) and (3), respectively).

(2) $$\mathrm{TNI}\;\left(\mathrm{kg}/d\right)=\frac{\mathrm{FE}\;\times \;\mathrm{CIF}}{\mathrm{CIFO}\;\times \;\mathrm{CN}}$$

(3) $$\mathrm{DN}\;\left(\%\right)=\frac{\mathrm{TNI}-\mathrm{FE}}{\mathrm{TNI}}$$

where TNI is total nutrient intake, FE is fecal excretion (kg/d), CIF is the concentration of the iNDF indicator in the feces (kg/kg), CFFO is the concentration of the iNDF indicator in the forage (kg/kg), and CN is the concentration of nutrients in the forage.

The supplement intake was evaluated by the difference between the quantity supplied and the feed leftover, with the daily supplementation of 0.3% BW established according to the animal weight and number of animals in the paddock.

### 2.4. Experiment 2: Animal Performance

The animals were distributed in a completely randomized design. The total experimental period was 105 d, and the animals were adapted to diets during the first 21 d, and the remaining 84 d were divided into three experimental periods of 28 d each.

In the MS condition, the animals received a mineral supplement *ad libitum*, and in the other conditions, the animals were supplemented at 0.3% BW every morning (11 a.m.). The supplements were allocated to an uncovered feeder, with a linear space of 0.3 m per animal. 

#### 2.4.1. Mass and Chemical Composition of Herbage

Considering the strong relationship between the herbage mass (HM) and pasture height (i.e., bulk density), we randomly measured 80 points/ha of height with a graduated ruler in each paddock. In order to determine the HM, every 28 d, four representative samples of the mean height of the paddock were collected by cutting all of the forage present in a 0.25 m^2^ circular frame, 5 cm from the soil [24].

Each sample was divided into two sub-samples, one for determination of total dry matter and the other for morphological composition by fraction of leaf, stem + sheath, and dead material. After drying in an oven at 55 °C for 72 h, the TDM and morphological components were obtained to estimate the HM and leaf-stem ratio [25]. To calculate the FA, the average HM was divided by the average BW of each paddock and period [26].

To evaluate the chemical composition of forage, 20 hand-plucked samples were randomly taken at the average forage height from each paddock during the last week of each period [27]. After weighing, the samples were dried at 55 °C for 72 h in a forced-air oven, and then ground in a Wiley mill with a 1 mm sieve. 

The chemical components of forage were analyzed according to the AOAC methodologies [28], as follows: Dry matter (DM, method 934.01); organic matter (OM, method 942.05); crude protein (CP) using LECO^®^ FP 528 (Leco Corporation, MI, USA); ether extract (EE); and gross energy through the complete combustion of the samples using an automatic bomb calorimeter. The protein fractions were determined by Licitra et al. [29] and the carbohydrate fractions by Sniffen et al. [30]. 

The neutral detergent fiber (NDF) of supplements was measured by adding alpha-amylase without the addition of sodium sulfite, whereas the NDF of forage was measured without any ecompounds. The neutral detergent fiber free of ash and protein (apNDF) and acid detergent fiber (ADF) analyses were performed using polyester filter bags and ANKOM^®^ equipment (Ankom Technologies, Macedon, NY, USA) [31]. 

#### 2.4.2. Animal Performance

To determine the average daily gain (ADG) of animals, weighing was performed at the beginning (initial body weight, IBW), before the adaptation period, and the end (final body weight, FBW) of the total experimental period, after a 14 h feed and water fast. In addition, every 28 d after the adaptation period, we conducted an intermediate weighing, with no fasting, in order to adjust the stocking rate, grazing height, and supplement quantity.

The gain per area was calculated according to the test ADG and the number of regulators and test animals of each paddock during the evaluation period [16]. The measurement of the total body weight of animals in each paddock and period allowed the estimation of the stocking rate in animal units per hectare (450 kg BW/ha).

#### 2.4.3. Enteric CH_4_ Emissions 

The evaluation of enteric CH_4_ emissions of 24 animals (six animals/treatment) was conducted during the last 6 d of the last two periods of the experiment. Methane emissions were measured using the sulfur hexafluoride (SF_6_) tracer gas technique, according to the manual of the Global Research Alliance for Greenhouse Gases on Agriculture [32]. 

First, the animals were submitted to a 7 d adaptation period for halter and PVC yoke use. A calibrated SF_6_ permeation tube with a mean of 90.0 μg/h release rate was orally introduced into the rumen/reticulum of each animal 8 d before the first collection. After the adaptation period, a collector-storage yoke of PVC (60 mm, class 20 pipe) was placed on the neck of the animal. 

To measure the background CH_4_ concentration, a blank collector set (yoke + halter) was installed in the paddocks. Enteric CH_4_ production was calculated using Equation (4).

(4) $${\mathrm{QCH}}_{4}=\frac{{\mathrm{QSF}}_{6}\times \left(\left[{\mathrm{CH}}_{4}\right]y-\left[{\mathrm{CH}}_{4}\right]b\right)}{\left[{\mathrm{SF}}_{6}\right]}$$

where QCH_4_ is the CH_4_ emission rate, QSF_6_ is the known rate of SF_6_ emission, [CH_4_]y is the CH_4_ concentration (ppm) of the collecting yoke, [CH_4_]b is the CH_4_ concentration of the blank yoke, and [SF_6_] is the concentration of SF_6_ (ppt) of the blank yoke.

After the adaptation period, gases were collected over 6 consecutive days every 24 h at 7 a.m. The concentrations of CH_4_ and SF_6_ were determined using a Shimadzu GC2014 gas chromatograph (Kyoto, Japan) equipped with flame ionization and micro-electron capture detectors. CH_4_ emissions were then expressed as g CH_4_/d, kg CH_4_/kg weight gain, g CH_4_/kg DM intake, g CH_4_/kg OM intake, and CH_4_ conversion rate (Ym). 

To estimate the digestible energy from the digestive percentage of the gross energy (Ym), we used Equation (5) [11,33], considering 0.05565 MJ/g CH_4_ and the energy obtained in the hand-plucked samples (EH).

(5) $$\mathrm{Ym}\;\left(\%\right)=\frac{\left({\mathrm{CH}}_{4}\times 0.05565\right)}{\mathrm{EH}}\times 100$$

### 2.5. Statistical Analysis

First, data were analyzed for homoscedasticity and normality of the residues using the PROC MIXED procedure of the SAS software [34]. To evaluate the ruminal parameters, intake, and digestibility of nutrients of the cannulated animals (Experiment 1), the experimental design was a double Latin square, with repeated time measurements (time of sample collection, h), using the AR-1 covariance matrix by the Akaike criterion. Ruminal parameters, supplement, Latin square, animal within square, and time of sample collection (h) were considered fixed effects, and animal, period, and residual error were considered random effects. For intake and digestibility, the time of sample collection (h) was not considered a fixed effect. In the presence of significant treatment effects, averages were compared using orthogonal contrasts, and Tukey’s test was used to compare the means of sample collection times (0, 2, 4, 6, and 8 h after supplementation) for ruminal parameters. The level of significance used to assess the differences between the means was α = 0.05. 

In Experiment 2, we used a completely randomized design, considering the paddocks as experimental units (three paddocks/treatment) for variables related to animal performance, and the animals for variables related to enteric CH_4_. Initial BW was used as a covariate for the statistical analysis of ADG and paddocks as random effects on animal performance. The animals were considered random effects for the variables related to enteric CH_4_. In the presence of a significant treatment effect, the averages were compared using orthogonal contrasts.

To compare the means of the variables between treatments in the two experiments, orthogonal contrasts were used to compare the means of the variables, as follows: MS vs. (CMS, 50DDG, and 100DDG) is the comparison of MS treatment versus treatments with supplements (CMS, 50DDG, and 100DDG); CMS vs. (50DDG and 100DDG) is the comparison of CMS versus treatments with inclusion of DDG (50DDG and 100DDG); and 50DDG vs. 100DDG is the comparison of 50DDG treatment versus 100DDG treatment. Differences were considered significant at *p* ≤ 0.05.

## 3. Results

### 3.1. Experiment 1

#### 3.1.1. Nutrient Intake and Digestibility 

The level of cottonseed meal replacement by DDG affected the total dry matter intake (*p* = 0.033), organic matter intake (*p* = 0.050), forage intake (*p* = 0.035), and digestible organic matter intake (*p* = 0.031), which were higher in 50DDG than 100DDG. The intake of TDN was higher in 100DDG than 50DDG (*p* = 0.027). The apparent digestibility of OM was not altered by the treatments (*p* > 0.05) (Table 4).

#### 3.1.2. Ruminal Parameters 

The pH, acetate, propionate, and acetate:propionate were influenced by the treatments (Table 5). Both the pH and acetate were higher in MS than the other treatments (*p* < 0.001). MS promoted the lowest average of propionate (17.81 mmol/L) compared to the other treatments (*p* = 0.013), and CMS promoted the highest average (18.51 mmol/L) compared to the treatments with DDG (*p* = 0.027). 

Comparing 50DDG and 100DDG, the concentration of propionate was higher in 50DDG (18.42 mmol/L; *p* = 0.003). The acetate:propionate ratio was higher in the MS treatment (4.04) than in the other treatments (*p* = 0.006). In addition, supplementation with DDG produced a higher acetate:propionate ratio than CMS (*p* = 0.044), and 100DDG produced the highest ratio (4.02), compared to 50DDG (3.87) (*p* = 0.007) (Table 5).

**Figure 1:**
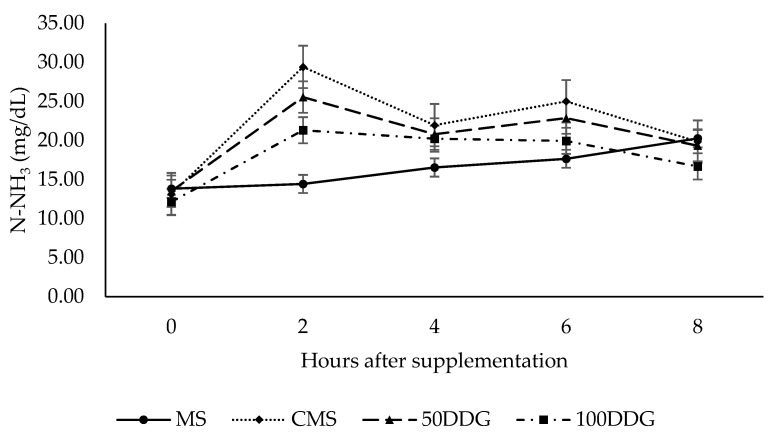
Ammoniacal nitrogen of cannulated young bulls fed with mineral supplementation (MS); conventional multiple supplement (energy/protein) with cottonseed meal and citrus pulp (CMS); supplement with 50% of CMS protein source replaced by DDG (50DDG); and supplement with 100% of CMS protein source replaced by DDG (100DDG), grazing Marandu grass during the wet season.

The interaction between the supplement and time of collection (*p* ≤ 0.05) was observed for N-NH_3_ (Figure 1). The average values of N-NH_3_ increased between 0 and 2 h after supplementation with supplements provided at 0.3% BW. After 2 h of supplementation, N-NH_3_ decreased, reaching values similar to the initial values after 8 h of supplementation. Supplementation with CMS promoted higher values of N-NH_3_ compared to the treatments with DDG. Additionally, the MS-animals’ maximum concentration of N-NH_3_ was seen 8 h after supplementation (20.28 mg/dL), whilst other supplements promoted the maximum concentration of N-NH_3_ 2 h after supplementation.

The pH, VFA, and acetate:propionate values differed between collection times (Table 6). The pH decreased after 4 h, with the lowest value observed at 8 h. The concentration of acetate was highest at 0 and 2 h, and lowest at 8 h, whilst the lowest concentration of propionate (17.11 mmol/L) was observed at time 0. The concentration of butyrate was similar between times 0, 2, 4, and 6 h, ranging from 10.20 to 10.77 mmol/L, and the highest concentration was observed at 8 h (11.47 mmol L/). At time 0, the acetate:propionate ratio was highest (4.27 mmol/L), reaching an intermediate value at 2 h (3.97 mmol/L) and lower values were observed after 4 h. 

### 3.2. Experiment 2

#### Animal Performance and Enteric CH_4_ Emissions

The FBW and ADG differed among the treatments (*p* ≤ 0.05) (Table 7), being greater for animals that received CMS, 50DDG, and 100DDG, compared to those that received MS (*p* = 0.011). The mean FBW was 439.7 kg, and ADG was 1.07 kg/d for the supplemented animals, representing an increase of 7.24% in FBW and 18.89% in ADG compared to the mineral supplementation. There were no differences in the stocking rate and gain per hectare (*p* > 0.05; Table 7).

The CH_4_ emission variables did not differ among the treatments (*p* > 0.05) (Table 8). The average enteric CH_4_ emissions were 136.6 g/d; 134.23 kg/kg weight gain; 16.00 g/kg DMI, and 26.25 g/kg OMI. 

Supplementation with a concentrate or mineral mixture did not affect enteric CH_4_ emissions of grazing animals or the CH_4_ conversion rate (*p* > 0.05) (Table 8). 

## 4. Discussion

### 4.1. Nutrient Intake and Digestibility

Both the nutritional and non-nutritional factors affect forage intake in grazing animals. Nutritional factors include the chemical composition and digestibility of the forage, whilst non-nutritional factors include forage allowance, animal selection, grazing time, sward structure, bit size, and rate [35,36]. Although supplements were provided at the same level (0.3% BW) and pastures were equally managed using the same strategies and level of fertilization, the animals that received 50DDG had a higher forage intake (kg/d) than 100DDG, but when converted to %BW, the forage intake was similar between both treatments. Meanwhile, the treatment with 50DDG also promoted greater TDMI than 100DDG, as verified by Hoffmann et al. [6]. 

One possible reason for the higher DM and forage intake in 50DDG compared to 100DDG is the supplement apNDF content, which was 312 and 372 g/kg, respectively. The intake is likely controlled by physical factors, such as the rumen fill capacity of the animal for fibrous feed, causing physical satiation [36,37]. Therefore, we propose that the animals that received a supplement with higher fiber content (100DDG) had quicker physiological satiety responses, thus reducing their intake. 

The nutrient intake is listed as the primary factor in feed conversion into the animal product. In this sense, the intake of the digestible fraction of a nutrient is affected more by its quantity than by the digestibility itself [38]. Nonetheless, the feed intake directly affects the passage rate through the digestive tract, influencing the digestibility of the ingested diet. However, even with greater OM, DOM, TDN, and TDMI intake, there were no differences in nutrient digestibility or ADG between the DDG treatments. 

Poppi and McLennan [39] proposed that the maximum efficiency of microbial protein synthesis and transfer of ingested protein to the intestine is achieved when the relationship is lower than 160 g CP/kg DOM. While values above 210 g CP/kg DOM result in reduced utilization of forage protein in the process of ruminal digestion and microbial protein synthesis, generating appreciable losses of N, as occurs in pastures fertilized with high doses of N. 

In this study, the relationship of g CP/kg DOM was similar among the treatments, all of which were higher than 210 g CP/kg DOM, especially in 100DDG, suggesting a possible low efficiency of protein utilization. In addition, Hoffmann et al. [8] found an average of 218.1 g CP/kg DOM in supplement treatments, also higher than the suggested value. However, the high proportion of DDG in the diet provided a high amount of energy, decreasing the forage intake and, consequently, the DOM intake arising from the forage, which resulted in greater relationship values. 

### 4.2. Ruminal Parameters 

The similarity of ruminal pH values of the supplemented animals in this study, which were lower than those of animals that received only the mineral mixture, indicates that the decrease in pH is influenced more by the level of supplementation than by the ingredients used in the formulation of supplements [40]. In addition, a reduction in ruminal pH values is expected with an increase in collection time after supplementation, due to the rapid fermentation of carbohydrates in the rumen and decreased salivation due to the shorter rumination time, resulting in a drop in pH [41]. This decrease in pH may also be associated with the peak concentration of ammonia in the rumen after feeding, as the reduced pH reduces the absorption of ammonia by the rumen and decreases microbial growth [42]. It is worth mentioning that the average ruminal pH values in this study were not reduced to below 6.2, both due to the treatment types and the collection times after supplementation, which may have favored the growth of cellulolytic bacteria [40] and thus contributed to fiber digestion.

Rumen N-NH_3_ values indicate that none of the treatments imposed limitations on the protein intake, since the minimum concentration of 10 mg/dL recommended by Leng [43] for adequate ruminal fiber fermentation was exceeded in all of the treatments. Microbial synthesis is maximized when the fermentation of dietary energy sources and protein degradation are synchronized [44,45,46]. Detmann et al. [47] reported that the ruminal N balance is positive when the concentration of N-NH_3_ is 9.7 mg dL, and it is maximized when the concentration exceeds 15.9 mg/dL, a value close to 15.33 mg/dL reported by Lazzarini et al. [48] necessary to maximize the DM intake of animals grazing tropical pastures. Concentrations of N-NH_3_ in the supplemented animals in this study were less than 15 mg/dL only at time 0, which indicates an ideal supply of N in the rumen for microbial development and protein synthesis.

The reduction in acetate over time after supplementation likely occurred as it is a substrate for the production of butyrate via the inverse β-oxidase pathway [49], which also explains the decrease in the acetate:propionate ratio. Nogueira et al. [50] observed a similar response in ruminal parameters of cows fed with cottonseed in the successive hours after supplementation (0 to 12 h), where acetate decreased between 3 and 6 h after supplementation, and propionate had a slight increase between 0 and 3 h, and after 3 h remained constant until 12 h after supplementation.

The higher DM intake observed in the animals that received 50DDG (Table 4), associated with the chemical composition of forage, may have caused the higher concentrations of propionate (18.42 mmol/L) seen, compared to 100DDG. The higher acetate:propionate ratio observed in the 100DDG treatment may be a consequence of the greater content of potentially degradable NDF (pdNDF) present in DDG [51]. 

The type of substrate fermented in the rumen has a direct influence on pH, microbial population, and fermentation products. Diets with a higher proportion of concentrate generate lower rumen pH, a favorable environment for amylolytic microorganisms, and an increase in the proportion of propionate [52]. Conversely, diets with a greater proportion of roughage resulted in higher ruminal pH and proportion of acetate. The supply of concentrated supplements for grazing animals can change the proportion of roughage and the concentrate of the total ingested diet, with supplemented animals receiving a higher proportion of concentrate when compared to the non-supplemented animals. This explains the greater proportion of propionate and lower proportion of acetate in the supplemented animals.

### 4.3. Animal Performance 

Supplementation of grazing beef cattle can result in greater intake of dry matter and nutrients. Consequently, increases in daily weight gain are observed when there is a balance between the protein and energy requirements [11,12]. In this study, supplementation with concentrate provided approximately 20% more ADG than the mineral supplementation. Nonetheless, there were no differences in ADG, stocking rate or GPH among the treatments. Pasture management should control the quality and quantity of the available forage. Therefore, the higher ADG seen in the supplemented animals is a response to the protein/energy supply [11,53]. 

In our study, the stocking rate was adjusted in all of the paddocks to maintain 25 cm of height and therefore, the forage mass was the same. The N fertilization and pasture management were also the same. However, the nutrient intake changed due to the nutrients provided by the supplement.

Another possible reason for the greater animal performance in animals supplemented with DDG is its protein profile, which contains high RUP content, which is responsible for the increase in essential amino acids and their metabolizable pool [54]. However, the replacement of cottonseed meal with DDG did not affect the productive responses, proving that it is possible to use this co-product as a cheaper protein source without influencing animal performance. 

### 4.4. Enteric CH_4_ Emissions 

Enteric CH_4_ emissions can be altered by a higher proportion of non-fibrous carbohydrates in the diet, since methanogenic microorganisms in the rumen produce CH_4_ from H_2_ and CO_2_ [55]. Therefore, supplementation tends to reduce the supply of H_2_ to the methanogenic population in the rumen and consequently decreases CH_4_ emissions [56]. However, in this study, there was no effect of supplementation on the emission of enteric CH_4_ compared to the animals that received the mineral mixture.

The similarity in nutritional conditions may have been responsible for the lack of differences in CH_4_ emissions. In addition, the results of this study corroborate those of Barbero et al. [11], in which pasture and supplementation management techniques were adopted in similar ways, thereby mitigating gas emissions.

As reported by Haque [57], there are two main causes for the variation in CH_4_ production: The amount of fermented carbohydrates in the rumen and the relative proportions of propionate and acetate produced. This highlights the importance of pasture management, with the aim to provide a forage supply with better nutritive value [58]. The use of concentrated supplements to provide a properly balanced diet has become an interesting strategy to increase the performance and weight gain by area, and reduce the enteric CH_4_ emissions of grazing beef cattle, which is favorable from both a productive and environmental point of view [59]. Although different ingredients were used in the formulation of the supplements, it is possible that the high proportion of forage due to the characteristics of the pasture contributed to the absence of differences in the emission of enteric CH_4_.

The CH_4_ conversion rate (Ym) was similar among the treatments and numerically followed the CH_4_ emissions (g/d) order, as it was calculated based on this value and the energy gross of hand-plucked and supplement samples. However, the average Yms were lower than that reported by Hoffmann et al. [8], who evaluated the same supplements and level of DDG replacement, but in lighter animals (255 ± 21 kg BW) and greater CH_4_ emissions per kg of dry matter intake. In addition, the results of MS, CMS, and 50DDG were below the range proposed by USEPA [60], between 5.5 and 6.5% in North America and Eastern Europe, and all of the treatments were below the IPCC [61] range, between 6.5 and 7.5% for grazing cattle under tropical conditions. This shows that Nellore bulls grazing high-quality Marandu grass pastures supplemented with a mineral mixture, cottonseed meal or DDG at 0.3% BW represent a lower loss of digestible energy ingested in the CH_4_ form.

## 5. Conclusions

Dried distillers’ grains are an alternative to the cottonseed meal, due to their capacity to provide the same animal performance and enteric CH_4_ emissions of grazing Nellore cattle during the rearing phase in the wet season, for a cheaper price. Both of the ingredients can be used to intensify beef cattle production in tropical pastures. Nonetheless, during this phase, supplementation with concentrates at 0.3% BW improved the productive performance of animals under grazing. However, further studies should be conducted considering the market availability and economic analysis of this co-product.

## Figures and Tables

**Table 1:** Ingredients and chemical composition of the four supplement strategies.

Item	Treatment			
	CMS	50DDG	100DDG	MS
*Ingredients (g/kg DM)*				
Cottonseed meal	322	165	-	-
Citrus pulp	562	496	402	-
DDG	-	215	446	-
Salt	36	36	36	-
Urea	31.5	31.5	31.5	-
Limestone	31	38	44	-
Monocalcium phosphate	14.9	17.3	19.5	-
Sulfur	2.0	-	19.3	-
Mineral premix ^1^	0.9	0.9	0.9	-
Monensin 200	0.4	0.4	0.4	-
Mineral salt ^2^	-	-	-	1000
*Chemical composition of supplements (g/kg DM)*				
apNDF	310	312	372	-
ADF	179	232	267	-
CP	226	229	213	-
EE	15	26	30	-
Starch concentration	18	17	16	-
TDN	607	625	630	-
GE (MJ/day)	182	164	151	-

MS: Mineral supplement; CMS: Conventional multiple supplement (energy/protein) with cottonseed meal and citrus pulp; 50DDG: Supplement with 50% of CMS protein source replaced by DDG; 100DDG: Supplement with 100% of CMS protein source replaced by DDG; apNDF: Neutral detergent fiber free of protein and ash; ADF: Acid detergent fiber; CP: Crude protein; EE: Ether extract; NFC: Non-fibrous carbohydrate; TDN: Total digestible nutrients; GE: Gross energy; ^1^ Guarantee levels: 14.0 g/kg Na; 21.7 g/kg Cl; 3.9 g/kg K; 2.4 g/kg Mg; 3.4 g/kg S; 277.1 mg/kg Fe; 52.7 mg/kg Cu; 57.4 mg/kg Mn; 189.1 mg/kg Zn; 4.5 mg/kg Co; 8.1 mg/kg I; 0.8 mg/kg Se; 60.0 mg/kg F. ^2^ Mineral salt: 153.0 g/kg Ca; 90.0 g/kg P; 125.0 g/kg Na; 10.0 g/kg Mg; 40.0 g/kg S; 1670.0 mg/kg Cu; 1500.0 g/kg Fe; 1290.0 mg/kg Mn; 6200.0 mg/kg Zn; 100.0 mg/kg Co; 124.0 mg/kg I; 32.0 mg/kg Se.

**Table 2:** Chemical components, carbohydrate and nitrogen fractionating of corn DDG and cottonseed meal.

Item	DDG	Cottonseed Meal
CP (g/kg DM)	288.50	389.92
NDF (g/kg DM)	659.31	368.07
ADF (g/kg DM)	243.89	228.65
EE (g/kg DM)	30.97	14.12
Carbohydrate fractions (g/kg DM)		
A + B1	148.60	453.20
B2	754.80	220.84
B3	96.60	325.96
Nitrogen fractions (g/kg CP)		
A	89.00	52.62
B1	78.50	144.73
B2	576.30	611.40
B3	74.90	106.54
C	181.30	84.73
RDP (g/kg DM)	486.00	742.73
RUP (g/kg DM)	514.00	257.27

DDG: Dried distiller’s grain; DM: Dry matter; CP: Crude protein; EE: Ether extract; NDF: Neutral detergent fiber; ADF: Acid detergent fiber; RDP: Rumen degradable protein; RUP: Rumen undegradable protein.

**Table 3:** Herbage mass (HM), morphological composition, herbage allowance (HA), and chemical composition of *Urochloa brizantha* cv Marandu pastures, during the wet season.

Variable	Treatment			
	MS	CMS	50DDG	100DDG
Herbage mass (kg DM/ha)	5660	5052	4897	4870
Leaf (g/kg DM)	330	350	380	340
Stem + sheath (g/kg DM)	380	350	350	340
Dead material (g/kg DM)	290	300	270	320
Leaf:stem ratio	0.80	1.0	1.1	1.0
Forage allowance (kg DM/kg BW)	2.09	2.16	2.10	2.13
*Chemical composition (g/kg DM)*				
OM	924	922	911	910
ApNDF	599	608	604	607
Indf	110	110	115	114
pdNDF	489	498	489	493
ADF	289	289	289	287
NFC	204	203	206	206
CP	126.7	128.1	125.3	126.7
*Protein fraction (% CP)*				
Fraction A	31.4	32.6	32.2	31.8
Fraction B1+B2	34.3	34.9	33.2	33.7
Fraction B3	27.8	26.7	27.5	27.8
Fraction C	6.5	5.8	7.1	6.7

MS: Mineral supplement; CMS: Conventional multiple supplement (energy/protein) with cottonseed meal and citrus pulp; 50DDG: Supplement with 50% of CMS protein source replaced by DDG; 100DDG: Supplement with 100% of CMS protein source replaced by DDG; BW: body weight; OM: Organic matter; apNDF: Neutral detergent fiber free of protein and ash; iNDF: Indigestible neutral detergent fiber; pdNDF: Potentially digestible neutral detergent fiber; ADF: Acid detergent fiber; NFC: Non-fibrous carbohydrate; CP: Crude protein.

**Table 4:** Nutrient intake and digestibility in cannulated young bulls fed with four supplementation strategies, grazing Marandu grass during the wet season.

Variable	Treatment				SEM	Contrast *p*-Value		
	MS	CMS	50DDG	100DDG		MS vs. (CMS, 50DDG, 100DDG)	CMS vs. (50DDG, 100DDG)	50DDG vs. 100DDG
TDMI (kg/day)	9.5	9.0	10.1	8.4	0.64	0.620	0.694	0.033
Forage intake (kg/day)	8.3	7.8	8.9	7.2	0.60	0.564	0.735	0.035
Forage intake (%BW)	2.5	2.3	2.7	2.1	0.10	0.103	0.405	0.498
OMI (kg/day)	7.3	7.0	7.9	6.5	0.53	0.795	0.670	0.050
DOMI (kg/day)	5.9	6.3	6.7	5.5	0.65	0.503	0.575	0.031
CPI (kg/day)	1.5	1.7	1.7	1.9	0.20	0.240	0.593	0.417
TDNI (kg/day)	6.5	6.1	6.9	5.8	0.92	0.092	0.075	0.027
OMD (g/kg)	624	704	666	668	53.2	0.108	0.301	0.960
g CP/kg DOM	254.2	269.8	253.7	345.5	32.32	0.477	0.583	0.386

MS: Mineral supplement; CMS: Conventional multiple supplement (energy/protein) with cot-tonseed meal and citrus pulp; 50DDG: Supplement with 50% of CMS protein source replaced by DDG; 100DDG: Supplement with 100% of CMS protein source replaced by DDG; TDMI: Total dry matter intake; BW: body weight; OMI: Organic matter intake; DOMI: Digestible organic matter intake; CPI: Crude protein intake; TDNI: Total digestible nutrient intake; OMD: Organic matter digestibility; DOM: Digestible organic matter; SEM: Standard error of the mean.

**Table 5:** Ruminal parameters of cannulated young bulls fed with mineral supplementation (MS); conventional multiple supplement (energy/protein) with cottonseed meal and citrus pulp (CMS); supplement with 50% of CMS protein source replaced by DDG (50DDG); and supplement with 100% of CMS protein source replaced by DDG (100DDG), grazing Marandu grass during the wet season.

Variable	Treatment					Contrast *p*-Value		
	MS	CMS	50DDG	100DDG	SEM	MS vs. (CMS, 50DDG, 100DDG)	CMS vs. (50DDG, 100DDG)	50DDG vs. 100DDG
pH	6.51	6.46	6.40	6.43	0.051	<0.001	0.073	0.225
Acetate (mmol/L)	71.50	70.66	70.81	71.48	0.503	0.049	0.177	0.155
Propionate (mmol/L)	17.81	18.51	18.42	17.79	0.382	0.013	0.027	0.003
Butyrate (mmol/L)	10.69	10.64	10.59	10.87	0.353	0.952	0.705	0.313
Acetate:propionate	4.04	3.85	3.87	4.02	0.104	0.006	0.044	0.007

SEM: Standard error of the mean.

**Table 6:** Ruminal parameters of cannulated young bulls grazing Marandu grass during the wet season over an 8 h period after supplementation.

Hours after Supplementation (h)	pH	Acetate (mmol/L)	Propionate (mmol/L)	Butyrate (mmol/L)	Acetate:Propionate
0	6.61 a	72.59 a	17.11 b	10.30 b	4.27 a
2	6.60 a	71.64 a	18.16 ab	10.20 b	3.97 ab
4	6.40 b	70.64 ab	18.61 a	10.74 b	3.81 b
6	6.41 b	70.89 ab	18.34 a	10.77 b	3.88 b
8	6.22 c	69.62 b	18.44 a	11.47 a	3.78 b
SEM	0.06	0.26	0.47	0.12	0.12

SEM: Standard error of the mean. Means in the same column followed by the same letter are not significantly different (*p* > 0.05) according to Tukey’s test.

**Table 7:** Performance, stocking rate, and gain per area of Nellore young bulls fed with mineral supplementation (MS); conventional multiple supplement (energy/protein) with cottonseed meal and citrus pulp (CMS); supplement with 50% of CMS protein source replaced by DDG (50DDG); and supplement with 100% of CMS protein source replaced by DDG (100DDG), grazing Marandu grass during the wet season.

Variable	Treatment				SEM	Contrast *p*-Value		
	MS	CMS	50DDG	100DDG		MS vs. (CMS, 50DDG, 100DDG)	CMS vs. (50DDG, 100DDG)	50DDG vs. 100DDG
IBW (kg)	334	351	344	352	5.11	0.137	0.590	0.280
FBW (kg)	410	439	431	449	7.90	0.011	0.930	0.140
ADG (kg/d)	0.90	1.04	1.04	1.15	0.06	0.042	0.480	0.240
Stocking rate (AU/ha)	6.54	6.07	5.76	6.00	0.36	0.190	0.700	0.700
GPH (kg BW/ha)	584	622	599	682	36.7	0.260	0.690	0.150

IBW: Initial body weight; FBW: Final body weight; ADG: Average daily gain; GPH: Gain per hectare; SEM: Standard error of the mean.

**Table 8:** Enteric CH_4_ emissions of young Nellore bulls fed with mineral supplementation (MS); conventional multiple supplement (energy/protein) with cottonseed meal and citrus pulp (CMS); supplement with 50% of CMS protein source replaced by DDG (50DDG); and supplement with 100% of CMS protein source replaced by DDG (100DDG), grazing Marandu grass during the wet season.

Variable	Treatment					Contrast *p*-Value		
	MS	CMS	50DDG	100DDG	SEM	MS vs. (CMS, 50DDG, 100DDG)	CMS vs. (50DDG, 100DDG)	50DDG vs. 100DDG
CH_4_ (g/d)	110.2	124.4	152.4	159.4	19.0	0.126	0.260	0.790
CH_4_ (kg/kg weight gain)	129.3	123.8	145.6	138.2	23.6	0.950	0.530	0.790
CH_4_ (g/kg DMI)	14.9	13.8	18.5	16.8	3.3	0.680	0.352	0.692
CH_4_ (g/kg OMI)	24.3	22.9	28.4	29.4	5.8	0.690	0.430	0.880
Ym (%)	4.10	4.17	5.05	6.39	0.691	0.126	0.260	0.790

CH_4_: Methane; DMI: Dry matter intake; OMI: Organic matter intake; Ym: CH_4_ conversion rate; SEM: Standard error of the mean.

## Data Availability

Data available on request due to restrictions (e.g., privacy or ethical).
